# Cervical impairments in subjects with migraine or tension type headache: an observational study

**DOI:** 10.3389/fneur.2024.1373912

**Published:** 2024-03-11

**Authors:** Jose Ángel del Blanco Muñiz, Alberto Sánchez Sierra, Arturo Ladriñán Maestro, Roberto Ucero Lozano, María Dolores Sosa-Reina, Daniel Martín Vera

**Affiliations:** ^1^Department of Physiotherapy, Faculty of Sport Sciences, Universidad Europea de Madrid, Villaviciosa de Odón, Madrid, Spain; ^2^Research Group on Exercise Therapy and Functional Rehabilitation, Faculty of Health Sciences, Universidad Europea de Madrid, Madrid, Spain; ^3^Faculty of Physiotherapy and Nursing, University of Castilla La Mancha, Toledo, Spain; ^4^Physiotherapy Research Group of Toledo (GIFTO), Faculty of Physiotherapy and Nursing, Universidad de Castilla-La Mancha, Toledo, Spain

**Keywords:** migraine, tension type headache, ultrasound, pain, functionality

## Abstract

**Objective:**

The aim of this investigation was to compare the thickness of the deep local muscles in the neck region, as well as local and widespread sensitivity and functionality, between individuals with migraine, Tension-Type Headache (TTH), and healthy controls. To date, we know that the onset of migraine and TTH share similar pathophysiological pathways. Nevertheless, there may be some anatomical and functional differences which would explain why clinicians may obtain variable results when treating both pathological entities with similar or equal approaches.

**Methods:**

An observational study was conducted in accordance with STROBE guidelines. The flexor longus colli and multifidus, two neck-stabilizing muscles, were measured using B-mode ultrasound imaging. The upper trapezius, masseter, temporalis, tibialis anterior, and median nerve all underwent bilateral pressure-pain threshold (PPT) assessments.

**Results:**

Ninety participants were enrolled in the study. All subjects were equally divided into TTH, migraine and control groups. The PPT values exhibited lower thresholds in patients with TTH than both migraine and healthy participants. Specifically, in the temporal muscle on both sides, patients with TTH exhibited a significantly lower threshold (*p* < 0.001)than both migraine and healthy participants. Patients with TTH displayed significantly lower thresholds in both upper trapezius muscles (right: *p* < 0.001; left: *p* = 0.001). Similar results were obtained for the tibialis anterior PPTs from both sides (*p* = 0.001 in both). However, both clinical groups exhibited lower thresholds than the healthy subjects (*p* < 0.001). In multifidus muscle cross-sectional area (CSA), no statistically significant differences were found between migraine patients and healthy subjects, both in relaxation and contraction (right; *p* > 0.05 and *p* > 0.05; left: *p* > 0.05 and *p* > 0.05). However, patients with TTH exhibited a smaller CSA than both migraine patients and healthy controls in multifidus relaxed and contracted state (right: *p* < 0.001 in both relaxed and contracted multifidus; left: *p* = 0.001 and *p* < 0.001, respectively). Similar results were obtained for the left longus colli muscle in both relaxation and contraction for patients with TTH and migraine compared with healthy subjects (*p* = 0.001 and *p* < 0.001, respectively, for muscle relaxation and *p* < 0.001 for muscle contraction). However, no significant differences were observed between patients with TTH and migraine (*p* < 0.05 in both relaxation and contraction). In the right longus colli, TTH and migraine patients had a significantly smaller CSA during contraction than healthy subjects (*p* < 0.001 in both comparisons). In the craniocervical flexion test, both groups of patients with TTH and migraine showed significantly lower values than healthy subjects (*p* < 0.001 in both comparisons). However, no significant differences were found between patients with TTH and migraineurs (*p* > 0.05).

**Conclusion:**

The findings provide a significant message for clinicians since anatomical and functional impairments were shown in patients with TTH and migraine. This study corroborates a lack of strength and smaller CSA in both clinical groups compared to controls. Therefore, strengthening programs may be addressed successfully for people with these pathological entities. To be more accurate, according to PPTs and CSA lower values in patients with TTH compared to migraine and controls, manual therapy approaches to desensitize craniocervical soft tissues and exercise therapy to increase endurance of deep cervical muscles may become meaningful especially in subjects with TTH. Nevertheless, in order to distinguish precisely between patients with TTH and migraine, different diagnostic strategies may be used in the future to describe these populations in further detail, which will assist health professionals in a more accurate treatment selection.

## 1 Introduction

According to recent studies, Tension-Type Headache (TTH) and migraine are the two most common types of primary headaches. According to a recent study published in 2019, an annual global prevalence of 38% is estimated to have these conditions in European countries (1). Migraine has a prevalence of approximately 12% worldwide, and 1%–2% of individuals suffer from its chronic form (2). Both types of headaches are more common in women than men, with a ratio of 3:1 (3).

Epidemiological studies on primary headaches face the challenge that their diagnosis is based solely on clinically agreed-upon data in the International Headache Society Classification (4).

The socioeconomic impact of the headaches is substantial. Stovner et al. estimated in 2016 that more than 3 billion people worldwide suffered from Migraine or TTH (5). An article published in The Lancet in 2017 highlighted headache as one of the leading causes of global disability, particularly in young and middle-aged women. Migraine is the second most common cause of disability after lower back pain (6). In Spain, the healthcare costs associated with caring for patients with headache raise to approximately 10 million euros annually (7).

While the primary management of primary headaches is predominantly pharmacological, there is growing evidence that physiotherapy or chiropractic treatments focus on the cervical spine, such as deep muscle strengthening exercises, to reduce the intensity and frequency of headache episodes, not only in the short term but also in the long term, in patients with TTH (8, 9) and individuals with migraine (10).

The association between headaches and cervical dysfunction has been increasingly accepted. Ashina et al. (11) stated that patients with headache have an incidence of neck pain from 2.5 to 6 times higher than individuals without headache.

In patients with TTH, a relationship with tension in the cervical musculature was established. Numerous studies have confirmed a higher proportion of subjects with alterations in the functioning of the cervical musculature compared with the healthy population. These patients may exhibit a higher coactivation level of antagonist musculature in isometric flexor-extensor strength tests, possibly because of altered motor patterns (12). Additionally, patients with TTH may have a reduced range of flexion-extension movement compared to healthy population (8) and notably increased pressure sensitivity (13).

Patients with migraine also demonstrate similar cervical dysfunctions. Benatto et al. (14) concluded that patients with migraine exhibited an altered flexor and extensor muscle strength ratio compared to healthy population and a decrease in strength in the craniocervical flexion test.

Furthermore, Luedtke et al. (15) aimed to quantify the presence of musculoskeletal dysfunction by conducting a total of 11 tests on 138 patients with migraine and 73 healthy patients. The study’s conclusion highlighted that migraine patients showed a higher presence of trigger points, reduced mobility in flexion and rotation of the upper cervical region, a decreased pain tolerance to palpation of the upper cervical joints and a lower activation of neck stabilizing muscles than healthy control subjects. Ninety-three percent of the subjects with headache showed any alterations in at least three exploratory tests.

The pathophysiological basis that seems to explain this relationship between headache and cervical dysfunction is the convergence of stimuli from cervical and cranial nociceptive receptors through the trigeminal nerve in the trigeminocervical complex (16).

Despite numerous publications concluded that there is a relationship between cervical musculoskeletal dysfunction and headache, the methodological quality is low, justifying further research. The primary objective of this study was to perform both an anatomical and functional assessment of the cervical musculature in subjects with migraine and TTH, as well as in healthy patients in order to determine if any differences appear between both clinical groups and healthy individuals, as well as between patients with TTH and migraine.

## 2 Methods

### 2.1 Study design and ethical considerations

This observational, cross-sectional study was carried out at the European University of Madrid following the recommendations of the Declaration of the Reporting Initiative on Observational Studies in Epidemiology (STROBE) (17), complied with the ethical criteria of the Declaration of Helsinki, and was approved by the ethical committee of the Rey Juan Carlos University of Madrid (ID: 1802202105721).

All participants were informed of the objectives and procedures and signed an informed consent form before conducting the study.

### 2.2 Participants

Ninety participants were recruited between workers and students from the Universidad Europea de Madrid (Villaviciosa de Odón) between January and March 2023, who fulfilled the following criteria:

a) Inclusion criteria:
  - Men and women aged 18–65 years old.
  - Symptomatic headache patients. These subjects must have been diagnosed by a specialist with chronic TTH or chronic migraine according to clinical criteria from The International Classification of Headache Disorders, 3rd edition (4).
  - Control subjects. Absence of headaches or migraine symptoms.
b) Exclusion criteria:
  - Pregnancy.
  - Central nervous system affection.
  - Medical treatment with botulinum toxin administration in the past 2 months.
  - Any chronic painful condition in the previous 6 months.

### 2.3 Procedure

Before admission, the patients received an information sheet detailing all testing procedures and were asked to provide informed consent. Patients received verbal information and had the opportunity to ask any questions to the researchers. Experienced investigators familiarized themselves with the protocol before patient enrollment began. To avoid flaws, the protocol description was checked by two different members of the research group.

### 2.4 Outcomes

Anthropometric variables were collected using Shepherd’s method, with particular attention to height, weight, and body mass index (BMI) measurements (18). Height and weight data were gathered using a tape measure and a numerical scale.

#### 2.4.1 Muscle thickness

**Figure 1:**
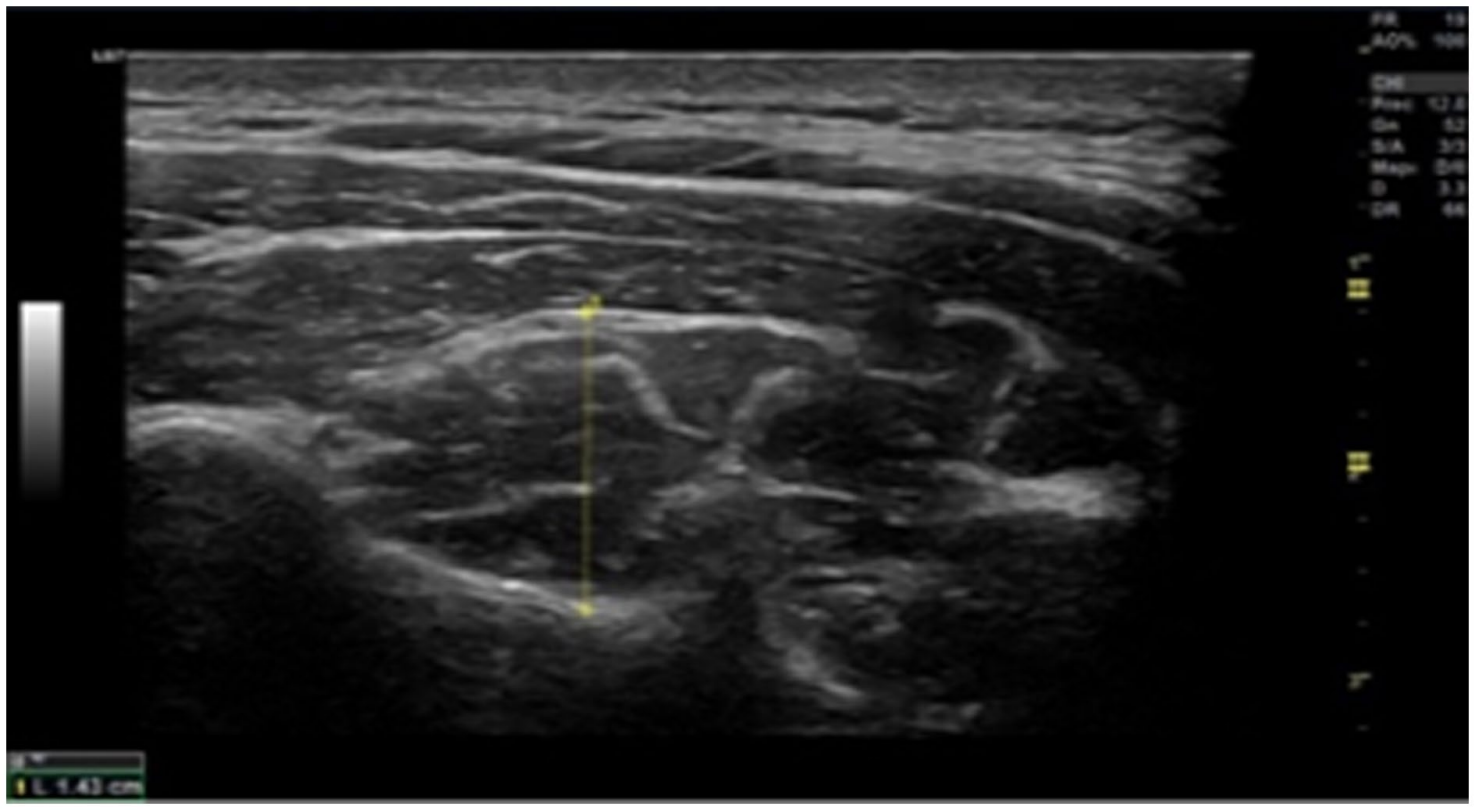
Cross-sectional image of a multifidus ultrasonography at C5. Up to the echogenic line of the hyperechoic fascia between the semispinalis cervicis and semispinalis capitis, the rater’s assessment of the muscle’s thickest point is where the probe is positioned 90° to the lamina of C5.

**Figure 2:**
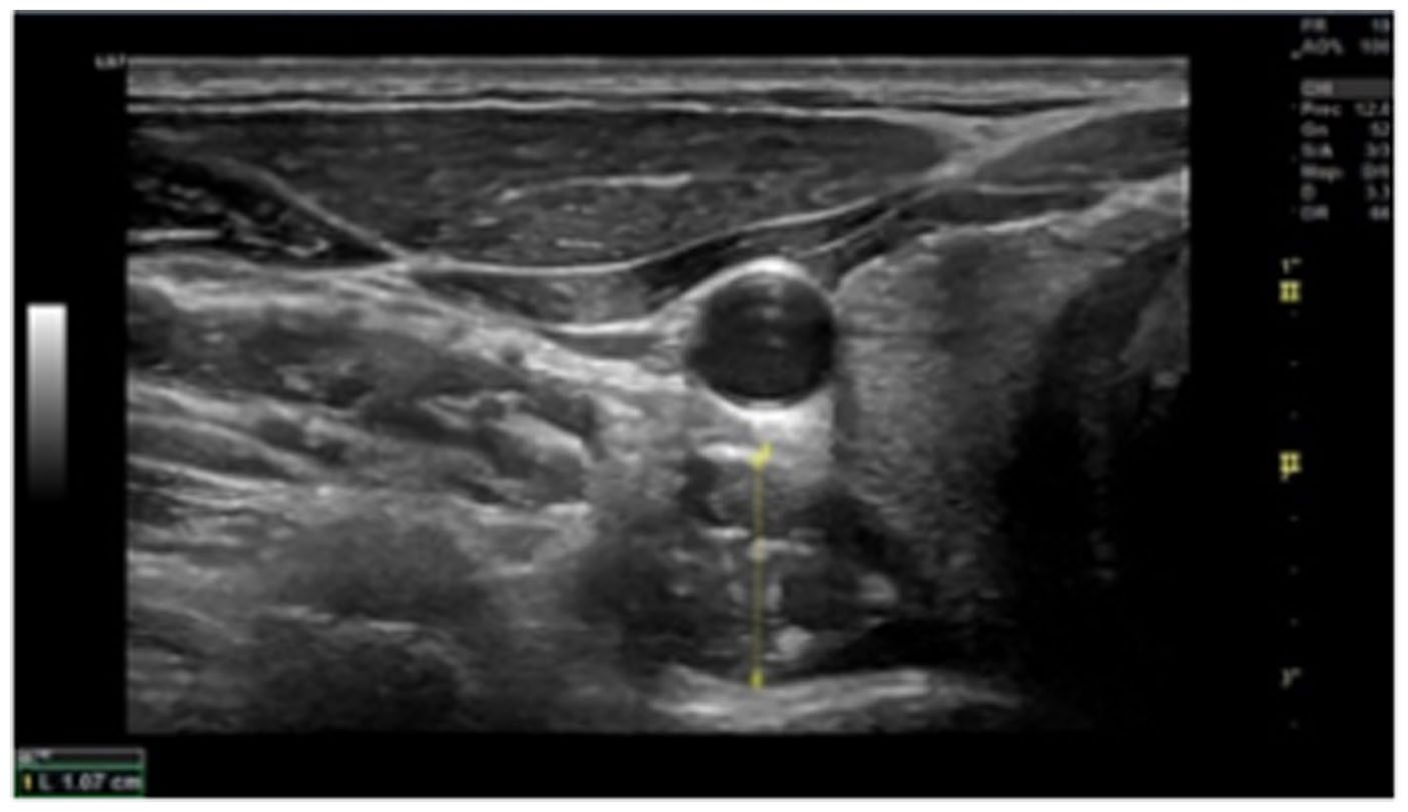
Ultrasound image of the longus colli at C6 (transverse view). The probe is positioned on the interface between the Lcol and the pre-fascial tissue encircling the carotid artery, as well as the midpoint of the ventral surface of the C6 vertebral body.

Ultrasonography was performed using a high-resolution device (GE Healthcare, Chicago, United States) to assess cross-sectional area (CSA). Deep local muscles evaluation was performed at the C5-C6 level (Figures 1, 2). Data were collected bilaterally at rest and during contraction of the multifidus cervicis and longus colli muscles (19). To ensure that the muscles were relaxed before measurements were taken at rest, the assessor used palpation to check for any signs of muscle stress while the patient was asked to remain relaxed. To measure muscle thickness in contraction, participants conducted either a “double chin” maneuver (chin tuck) or neck extension, depending on the muscle being measured. This procedure was originally demonstrated by researchers to help patients to become familiar with it.

#### 2.4.2 Pressure pain threshold

The protocol described by Fleckenstein (20) was used in this study. A FORCE DIAL FDK/FDN 100 algometer (Wagner Instruments, Greenwich, CT, United States) was used as the accurate measurement device. PPT has shown the highest reliability as a method for determining the mechanical threshold (21). The participants were examined in a relaxed supine position. Bilateral PPT recordings of the masseter, temporalis, upper trapezius, tibialis anterior, and median nerves were collected. The specific points are as follows.

- Trapezius: Midpoint between C7 and acromion.
- Masseter: Jaw angle. 1 cm superior to the insertion point of the superficial layer.
- Temporalis: Mid fibers in the bone depression around 2 cm lateral to the outer edge of the eyebrow.
- Tibialis anterior: 5 cm down and 2 cm lateral to the anterior tibial tuberosity on the muscle belly.
- Median nerve: in the flexion crease of the elbow, medial to the biceps brachii tendon.
- Precise points were marked for reproducibility, and measurements were performed three times. The algometer was set on each spot, increasing the pressure at a rate of approximately 1 kg/cm^2^ per second.

The patients were familiarized with the recording method. The subjects were asked to mark when they first felt a painful sensation when applying pressure with the algometry device. PPT measures using an algometry device exhibited good to excellent inter-rater reliability (ICC: 0.64–0.92) and test–retest reliability (ICC: 0.72–0.95).

#### 2.4.3 Craniocervical flexion test (CCFT)

CCFT was used in accordance with Thongprasert and Kanlayanaphotporn’s technique (22) to evaluate the performance of local muscles in the cervical region using the Stabilizer Pressure biofeedback equipment (Chattanooga Group, Hixon, TN, United States). The subjects remained in a neutral neck posture while assuming a supine position with semiflexed knees. The stabilizer was initially inflated to 20 mmHg after being placed in the suboccipital region. Nodding was used to encourage the subjects to undertake craniocervical flexion, while the pressure was increased progressively by 2 mmHg from 20 to 30 mmHg. Each increase was maintained for 10 s. This method enabled the examiner to gauge the subject’s endurance capacity for the anterior deep cervical muscles (flexor longus colli) by assessing how long the subject could maintain the contraction without showing any fatiguing signs.

Fatigue indicators were tracked by palpation of superficial neck flexor muscles. CCFT was used to generate the performance index (PI) and activation score (AS). The highest pressure level that the individuals could reliably withstand for 10 s was denoted as AS. The number of times the test could be repeated at the AS was used to construct the PI, which measures the endurance capability of the deep cervical flexor muscles. The highest possible PI score of 100 was determined (10 repetitions at 10 mm Hg AS). Based on previous research, a scoring system was applied to the data, in which an abnormal reaction was defined as ≤4 mmHg for AS and ≤20 points for Pl. The 95% confidence interval was between 0.70 and 0.94, while the inter-rater reliability ICC value was 0.89. The intra-rater reliability ICC score was 0.87, with a 95% confidence interval of 0.77 and 0.93. The stabilizer device demonstrated excellent to very good intra-and inter-rater reliability (23).

### 2.5 Sample size and data collection methods

The sample size was calculated using G*Power V.3.1.9.7, with an effect size of 0.35, a power of 0.80, and a statistical significance of 95% (α = 0.05). Using these parameters, the minimum number of subjects considered was 82. Ultimately, the study included a total of 90 subjects, with 30 participants equally divided into chronic migraine, TTH, and control groups. A physical examination was conducted by a single examiner and self-reported forms were used to gather data. The corresponding author may provide the datasets used and examined in this study upon justifiable request.

### 2.6 Statistical analysis

Statistical analysis was performed with the SPSS v29 program. The chi-square test of independence was used to analyze the categorical variables. The data distribution of all quantitative variables in this study was initially assessed using the Shapiro–Wilk test. These results indicate that the data distribution was not normal. Therefore, they were analyzed using the Kruskal-Wallis test to compare multiple independent groups and its subsequent *post hoc* test with Bonferroni adjustment.

## 3 Results

### 3.1 Characteristics of the included subjects

In this study, 90 subjects participated, including 30 migraine patients, 30 tension-type headache patients, and 30 healthy individuals. Anthropometric data were collected from both groups (Table 1). The results revealed no statistically significant differences in sex, weight, or height (*p* > 0.05) between the groups. Nonetheless, discernible statistical distinctions in age were manifested among patients diagnosed with migraine and those experiencing TTH, revealing that the latter cohort exhibited a statistically significant older age profile than migraine sufferers (*p* = 0.007).

**Table 1:** Characteristics of the participants.

		Healthy controls (*n* = 30)	Migraine (*n* = 30)	TTH (*n* = 30)
Gender	Male *n* (%)	13 (43.3%)	10 (33.3%)	10 (33.3%)
	Female *n* (%)	17 (56.7%)	(20 /66.67%)	(20 /66.67%)
Age *(years)*		29 [46–23]	28 [43–23]	37.5^*^ [48–35]
Height *(meters)*		1.67 [1.75–1.60]	1.67 [1.74–1.61]	1.66 [1.74–1.62]
Weight *(kilograms)*		68 [74–57]	63 [71–61]	65 [70–60]

Data are presented as absolute or relative frequency or as median and interquartile range [75th percentile-25th percentile]. ^*^p < 0.05 compared to migraine group. TTH, Tension-type headache.

### 3.2 Pain pressure threshold

In the analysis of pain pressure thresholds across various muscle groups and nerves, distinct patterns emerged. Patients with TTH consistently displayed lower thresholds compared to both migraine patients and healthy subjects.

Specifically, in the right temporal muscle, patients with TTH exhibited a significantly lower threshold than healthy controls and migraine patients (3 [3.2–2.8] vs. 5.7 [5.9–5.3]; *p* < 0.001 and 3.65 [4.5–2.9]; *p* = 0.029, respectively). Migraine patients also demonstrated a lower threshold compared to healthy subjects (3.65 [4.5–2.9] vs. 5.7 [5.9–5.3]; *p* < 0.001). Similar trends were observed in the left temporal region, with patients with TTH showing lower thresholds than both healthy subjects and migraine patients (2.95 [3.1–2.7] vs. 5.6 [6–5.3] and 3.45 [4.7–2.8]; *p* < 0.001 in both comparisons), and migraine patients exhibiting a lower threshold compared to healthy subjects (3.45 [4.7–2.8] vs. 5.6 [6–5.3]; *p* < 0.001).

Moving to the upper trapezius muscles, patients with TTH displayed significantly lower thresholds on both sides compared to healthy subjects and migraine patients (right: 3.5 [3.8–3.3] vs. 6.6 [7–5.9] and 4.45 [5.1–3.8]; *p* < 0.001 in both comparisons; left: 3.5 [3.8–3.4] vs. 6.5 [7–6]; *p* < 0.001 and 4.15 [5–3.6]; *p* = 0.001). Migraine patients, in turn, demonstrated lower thresholds compared to healthy subjects (right: 4.45 [5.1–3.8] vs. 3.5 [3.8–3.3]; left: 4.15 [5–3.6] vs. 3.5 [3.8–3.4]; *p* < 0.001 in both comparisons).

Contrarily, no statistically significant differences were observed in the pain pressure thresholds of the masseter muscles between TTH and migraine patients (right: 2.4 [2.6–2.3] vs. 2.795 [3.2–2.2] and left: 2.5 [2.6–2.4] vs. 2.685 [3.03–2.3]; *p* < 0.05 in both comparisons). However, both patient groups exhibited lower thresholds compared to healthy subjects (right: 2.4 [2.6–2.3] and 2.795 [3.2–2.2] vs. 4.35 [4.7–4.2] and left: 2.5 [2.6–2.4] and 2.685 [3.03–2.3] vs. 4.4 [4.6–4.1]; *p* < 0.001 in all these comparisons).

Similarly, no statistically significant differences were found in the pressure pain thresholds of the median nerve between TTH and migraine patients (right: 3.6 [3.7–3.4] vs. 3.75 [4.1–3.3]; and left: 3.5 [3.7–3.3] vs. 3.9 [4.2–3.2]; *p* > 0.05 in both comparisons). However, both patient groups exhibited lower thresholds compared to healthy subjects (right: 3.6 [3.7–3.4] and 3.75 [4.1–3.3] vs. 6.35 [6.6–6]; and left: 3.5 [3.7–3.3] and 3.9 [4.2–3.2] vs. 6.35 [6.6–6]; *p* < 0.001 in both comparisons).

**Figure 3:**
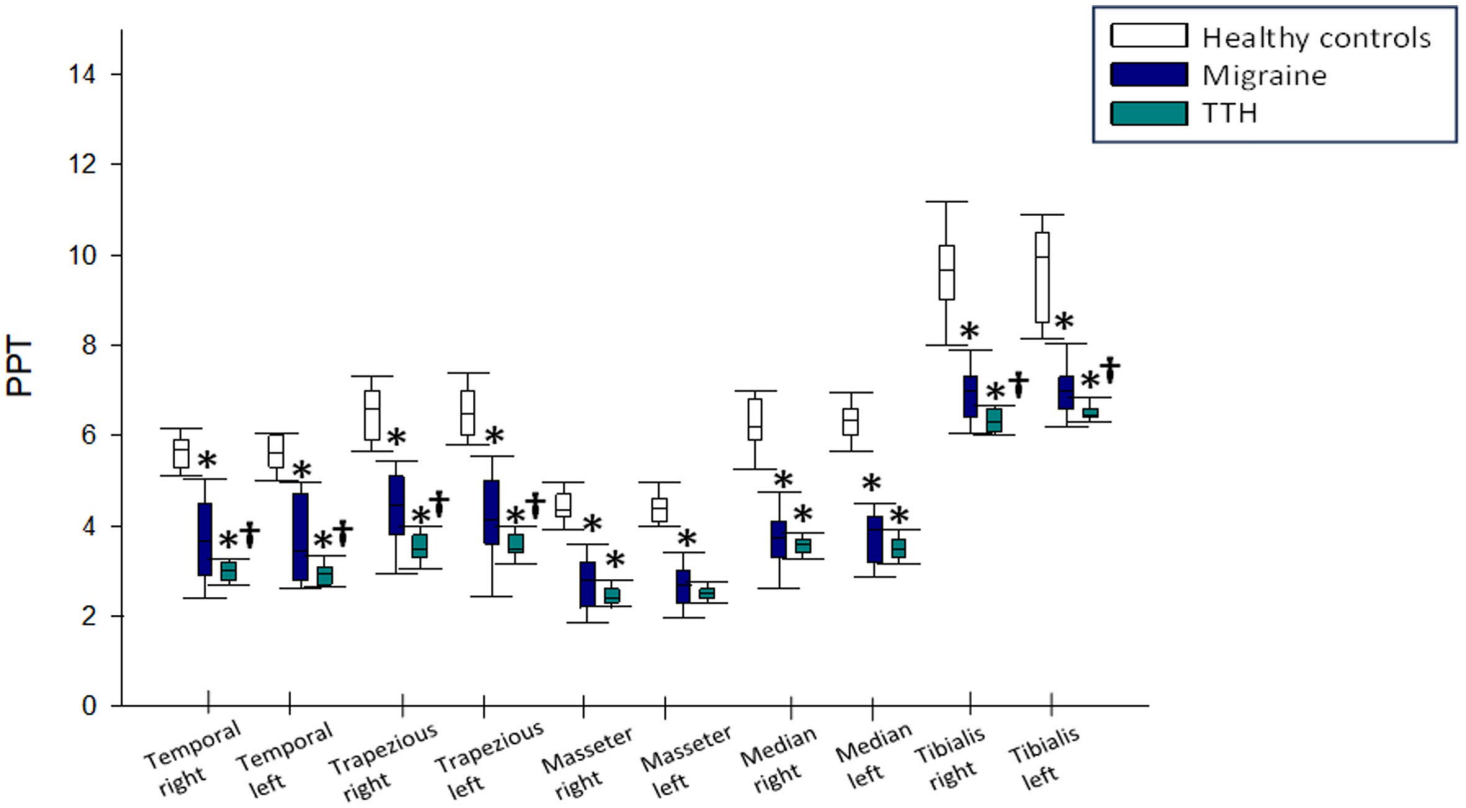
Results of pain pressure threshold in tension-type headache and migraine patients compared to healthy subjects. TTH, tension-type headache. ^*^p < 0.05 compared to healthy subjects. ^†^*p* < 0.05 compared to migraine patients.

In the assessment of the pain pressure threshold in the tibialis anterior, patients with TTH exhibited lower thresholds on both sides compared to both migraine patients and healthy subjects (right: 6.3 [6.6–6.1] vs. 7 [7.3–6.4]; *p* = 0.001 and 9.65 [10.2–9]; *p* < 0.001, respectively; left: 6.45 [6.6–6.4] vs. 7 [7.3–6.6]; p = 0.001 and 9.95 [10.5–8.5]; *p* < 0.001, respectively). Furthermore, migraine patients exhibited lower thresholds compared to healthy subjects in both tibialis anterior (right: 7 [7.3–6.4] vs. 9.65 [10.2–9]; and left: 7 [7.3–6.6] vs. 9.95 [10.5–8.5]; *p* < 0.001 in both comparisons; Figure 3).

### 3.3 Muscle thickness

**Figure 4:**
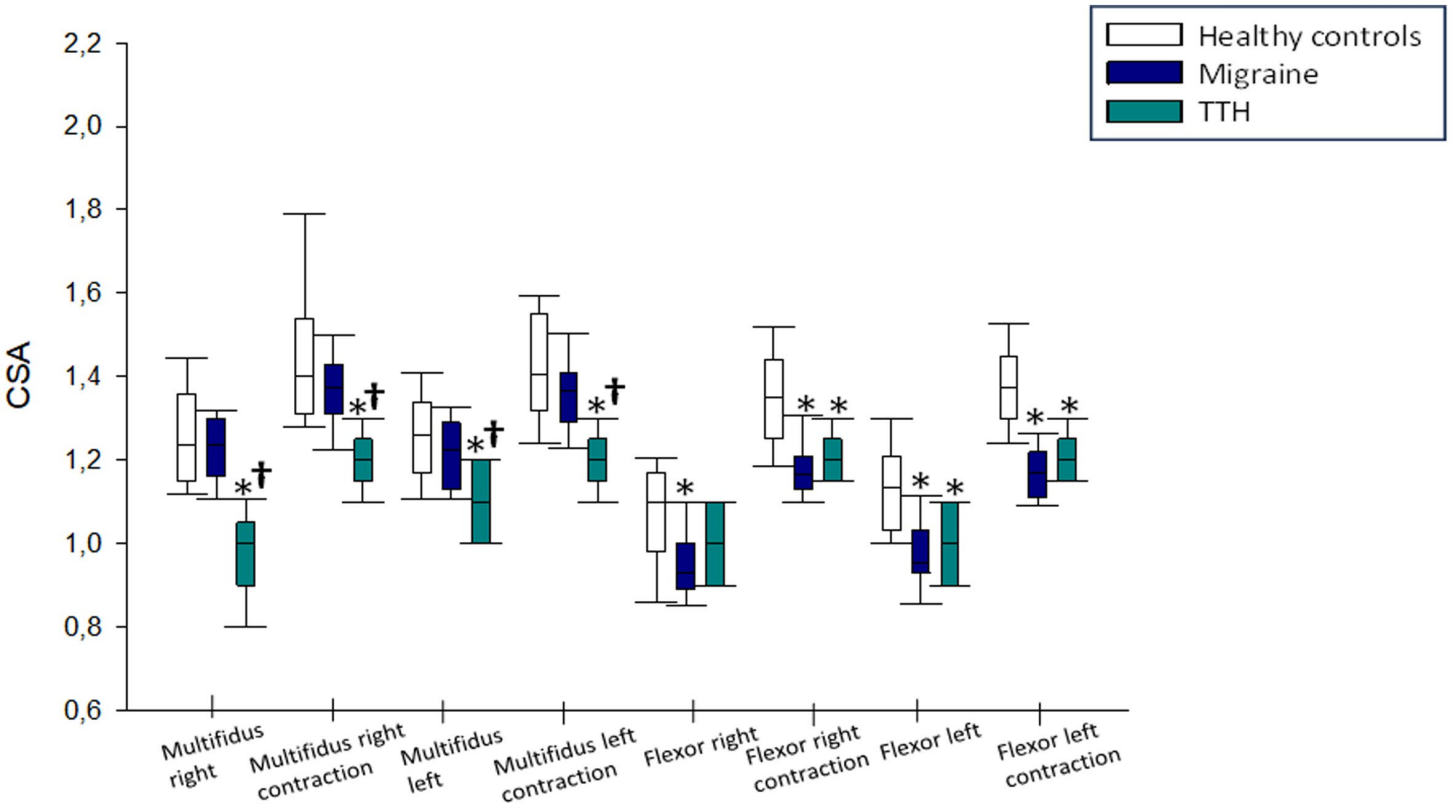
Results of cross-sectional area in tension-type headache and migraine patients compared to healthy subjects. TTH, tension-type headache. ^*^p < 0.05 compared to healthy subjects. ^†^*p* < 0.05 compared to migraine patients.

In the analysis of multifidus muscle cross-sectional area, no statistically significant differences were found between migraine patients and healthy subjects, both in relaxation and contraction (right: 1.235 [1.36–1.15] vs. 1.235 [1.3–1.16] and 1.375 [1.43–1.31] vs. 1.4 [1.54–1.31]; left: 1.225 [1.29–1.13] vs. 1.26 [1.34–1.17] and 1.365 [1.41–1.29] vs. 1.42 [1.58–1.32]; *p* > 0.05 in all these comparisons). However, patients with TTH exhibited a smaller cross-sectional area than both migraine patients and healthy controls, both in relaxation and contraction (right: 1 [1.05–0.9] vs. 1.235 [1.3–1.16] and 1.2 [1.25–1.15] vs. 1.375 [1.43–1.31]; *p* < 0,001 in both comparisons; left: 1 [1.1–0.9] vs. 0.955 [1.03–0.93]; *p* = 0.001 and 1.2 [1.25–1.15] vs. 1.17 [1.22–1.11]; *p* < 0.001, respectively). No significant differences were observed between patients with TTH and migraine patients as well as healthy subjects in the right longus colli muscle in relaxation (1 [1.1–0.9] vs. 0.93 [1–0.89] and 1.1 [1.17–0.98]; *p* > 0.05 in both comparisons). Nevertheless, TTH and migraine patients demonstrated a significantly smaller area in the same muscle in contraction compared to healthy subjects (1.165 [1.21–1.13] and 1.35 [1.44–1.25] vs. 1.35 [1.44–1.25]; *p* < 0.001 in both comparisons). Regarding the left longus colli muscle in both relaxation and contraction, TTH and migraine patients showed a smaller cross-sectional area than healthy subjects (1 [1.1–0.9] and 0.955 [1.03–0.93] vs. 1.35 [1.21–1.03]; *p* = 0.001 and p < 0.001, respectively in muscle in relaxation and 1.20 [1.25–1.15] and 1.17 [1.22–1.11] vs. 1.375 [1.45–1.30]; *p* < 0.001 in both comparisons regarding muscle in contraction). However, no statistically significant differences were observed between patients with TTH and migraine patients in this regard (left muscle in relaxation: 1 [1.1–0.9] vs. 0.955 [1.03–0.93] and left muscle in contraction: 1.2 [1.25–1.15] vs. 1.17 [1.22–1.11]; *p* > 0.05 in both comparisons; Figure 4).

### 3.4 Craniocervical flexion test

In craniocervical flexion test (CCFT), both TTH and migraine patients showed significantly lower values compared to healthy subjects (22 [22 − 20] and 20 [22 − 20] vs. 24 [24 − 22]; *p* < 0.001 in both comparisons). However, no statistically significant differences were found between TTH and migraine patients (22 [22 − 20] and 20 [22 − 20]; *p* > 0.05).

## 4 Discussion

The main focus of this investigation was to contrast healthy population with migraine and patients with TTH in terms of tissue mechanosensitivity of head and neck areas, as well as non-local tissues that may show central sensitization, morphology of the deep cervical muscles, and neck muscles functionality.

PPT has proven to be an effective variable for measuring mechanical pain thresholds in musculature, including the craniocervical muscles (24). This investigation results demonstrated a reduction in thresholds for TTH headache patients compared to the migraine group and healthy subjects in the temporal muscle, upper trapezius, and tibialis anterior. For other locations, no significant differences were observed between the pathological groups, but distinctions were evident compared to the healthy subject group. These findings align with previous reviews that also identified similar differences in PPT between population groups similar to ours (25). These intergroup differences may be attributed to alterations in central pain processing pathways from both pathological groups (26).

Significant differences in muscle thickness were observed between the pathological groups and the group of healthy subjects in this study. Previous studies have demonstrated the association between TTH and a reduction in muscle thickness in the multifidus and longus colli muscles (27). Positive correlations were found between craniocervical flexion test (CCFT) performance and multifidus thickness, along with negative correlations between deep cervical muscles thickness and pain intensity in these patients (28).

According to strength values, results revealed a substantial decrease in strength (*p* = 0.000) in both pathological groups compared to healthy volunteers, but no significant differences between the two groups with symptoms were shown. These results are consistent with previous studies in which muscle strength is also correlated with headache (29), and where no differences in muscle strength are evident between patients with migraine and tension-type headache (30). These outcomes align with similar studies that demonstrate reduced strength in flexor muscles of the neck region and forward head displacement in individuals with headache symptoms (30, 31). Patients experiencing non-specific neck pain, with or without a referred headache pattern, exhibit a direct correlation with a decrease in CCFT scores, showing higher reliability for the results of the Performance Index (PI) version of the test (32). While CCFT results can differentiate healthy volunteers from migraine sufferers, no distinction can be made with patients with TTH or individuals with neck-related disorders. Consequently, this assessment tool lacks sufficient discriminative validity among individuals with headache symptoms of diverse origins (33).

As both pathological conditions involve the trigeminovascular system, serving as a cornerstone to explain shared features (34), it is reasonable that CCFT may not reveal any significant difference. Additionally, the coexistence of both clinical entities makes accurate diagnosis challenging. However, recent research has identified differences in gray matter volume between migraine and TTH, with a more pronounced decrease in prefrontal and cerebellar areas in migraine compared to TTH (35). Further investigations are needed to guide clinicians in selecting the most effective functional diagnostic tools for high-quality tailored treatments.

## 5 Conclusion

This study demonstrates that patients with TTH and chronic migraine have more musculoskeletal and functional abnormalities than do healthy individuals. Among these, morphological changes were noted in all posterior and anterior deep cervical muscles of both migraineurs and patients with TTH, showing a larger atrophy compared to healthy population. These findings are accompanied by a reduction in the functional performance of anterior cervical muscles. Reduced PPT values are associated with higher levels of local and widespread pain sensitization, especially in patients with TTH, although migraine sufferers also displayed lower values than controls. As a result, both TTH and migraine patients may have compromised endogenous descending inhibitory pain modulation pathways, just as patients with other chronic pain states.

### 5.1 Clinical implications

The results of the study help clinicians to select better tailored treatment actions for individuals with TTH and migraine, especially targeting strengthening programs that improve both anatomy and function related variables. Nevertheless, in order to make a distinction between TTH and migraine patients, different diagnostic strategies may be used in the future to describe in further detail these populations, which will assist health professionals in a more accurate treatment selection.

## Data availability statement

The raw data supporting the conclusions of this article will be made available by the authors, without undue reservation.

## Ethics statement

The studies involving humans were approved by Ethical Committee of the Rey Juan Carlos University of Madrid (Spain). The studies were conducted in accordance with the local legislation and institutional requirements. The participants provided their written informed consent to participate in this study.

## Author contributions

JB: Conceptualization, Data curation, Formal analysis, Investigation, Methodology, Project administration, Supervision, Validation, Writing – original draft, Writing – review & editing. DM: Conceptualization, Data curation, Investigation, Methodology, Project administration, Supervision, Validation, Visualization, Writing – review & editing. AL: Conceptualization, Formal analysis, Methodology, Resources, Supervision, Validation, Visualization, Writing – review & editing. RU: Conceptualization, Data curation, Formal analysis, Investigation, Methodology, Software, Writing – review & editing. MS-R: Conceptualization, Formal Analysis, Investigation, Methodology, Software, Supervision, Validation, Visualization, Writing – original draft. AS: Conceptualization, Methodology, Project administration, Resources, Supervision, Visualization, Writing – original draft, Writing – review & editing.
